# Sanitary Inspectors and Public Funds

**Published:** 1906-09-29

**Authors:** 


### Sanitary Inspectors and Public Funds.

One would imagine that with long years of hygienic instruction and a multitude of sanitary commissions designed and formed with the avowed intention of instructing the public in the way to avoid such diseases as are due to want of sanitary method would be greatly on the decrease. This, however, is not the case. From the statement dealing with Metropolitan pauperism issued by the Local Government Board last week it appears the number of patients in fever and small-pox hospitals amounted to 3,946. In 1903 the corresponding number was 2,630; in 1904, 2,885; in 1905, 3,866; the acme being the figures of this year, set forth above. There is only one deduction from the results. The means at the disposal of the public must either be neglected or the departments of Local Government which are concerned with the public health must be in the hands of incapable local authorities. In some country districts the administration of the Public Health Acts is almost a dead letter. The medical officer is not only powerless to enforce his recommendations, but often holds his office at the will and pleasure of the very persons who are opposed to sanitation, or of the proprietors of nuisances which it is his duty to condemn. The public remain complacently ignorant on all medical questions, but nowhere is this more pronounced than in rural districts enjoying what is called "Local Government." Those that are under this rule refuse to accept the responsibility of governing themselves. The streets are unclean and undrained, and the scavenging is conducted on the principles adopted by Mrs. Maclarty. It would hardly be an exaggeration, judging from the experience of the past half-century in towns, to say that one-half of the mortality of rural England is preventible, and that its increase is almost solely due to the ignorance and apathy of local authorities.

Another contributory cause is the frivolous manner in which disinfectants are selected and their use ridiculed by the average sanitary inspector. As a rule he has but little training in the matters with which he is so closely concerned. He is the appanage of busy-bodies vested with a little brief authority who affect to scorn and depreciate the remedies and methods placed at their disposal. They are appointed usually for their loquacity, which is generally in direct ratio with their incompetency. One sanitary inspector in charge of a large urban district successfully runs a barber's shop. His interest in cleanliness would for that reason be assumed to be of a high order. Yet that gentleman on a recent occasion expressed the opinion that "smell and price were the only points that called for consideration in the choice of a disinfectant." If those clients of his who came to be tonsured understood the fundamental rules of cleanliness and the precautions which ought to be taken against the infectious diseases which a barber holding such views on disinfection and sterilisation is capable of scattering broadcast among them, this tonsorial sanitary inspector might be induced to change his views, and Local Government, so far as he was concerned, would assume a different aspect. It is a standing disgrace to Local Government that such officials should be entrusted with the spending of public money and with the power of selecting a disinfectant for public use. It is no uncommon thing to hear a sanitary inspector say, "We use no disinfectants here; the drains are perfect," as if perfect drainage will prevent the development of typhoid fever in a king's palace or in a baronial mansion, much less in the inefficient cloacas that sometimes pass for drains in rural and urban sanitary districts. Another sanitary inspector maintains that in his district "soap and water is good enough for us. We do not believe in disinfectants." Even where the argument in favour of the efficiency of disinfectants is admitted the inspector will urge that he uses the cheapest because when strong and efficient disinfectants are purchased the workmen who employ them persist in using the same quantity. Expenditure of public moneys should not be entrusted to sanitary inspectors such as these. It is, of course, needless to add that there are many sanitary inspectors who possess and act upon a knowledge of the bactericidal control of disinfectants and who are well equipped when dealing with problems of practical disinfection. But we urge that such subjects form no part of the sanitary inspector's education, and they should be left to be dealt with solely by the medical officer of health, who alone has the scientific training necessary to enable him to exercise that control over public expenditure for sanitary purposes—a control which is so markedly absent even in many of our largest cities.

